# Retinoic acid signalling in gastrointestinal parasite infections: lessons from mouse models

**DOI:** 10.1111/j.1365-3024.2012.01364.x

**Published:** 2012-07

**Authors:** R J M Hurst, K J Else

**Affiliations:** The University of ManchesterManchester, M13 9PT, UK

**Keywords:** gastrointestinal, helminth, retinoic acid, vitamin A

## Abstract

Retinoic acid or vitamin A is important for an extensive range of biological processes, including immunomodulatory functions, however, its role in gastrointestinal parasite infections is not yet clear. Despite this, parasite infected individuals are often supplemented with vitamin A, given the co-localised prevalence of parasitic infections and vitamin deficiencies. Therefore, it is important to understand the impact of this vitamin on the immune responses to gastrointestinal parasites. Here, we review data regarding the role of retinoic acid signalling in mouse models of intestinal nematode infection, with a view to understanding better the practice of giving vitamin A supplements to worm-infected people.

## Introduction

Retinoic acid signalling is known to be important for a wide variety of physiological processes, including immunomodulatory functions, though its role in immune responses to parasitic infections is not yet fully understood. The precursor of this metabolite, vitamin A or retinol, is an essential nutrient obtained from the diet, and deficiency in this vitamin can lead to an increased susceptibility to infectious diseases (1). Unfortunately, vitamin A deficiency is a public health problem in over 120 countries, affects 140 million pre-school children (2) and is responsible for approximately 1 million childhood deaths each year (3). Therefore, children in developing countries are often supplemented with vitamin A to prevent mortality and diarrhoea (4).

Interestingly, vitamin A deficiency and parasitic infections in human populations have extremely similar geographical distributions, with the same individuals experiencing both conditions (5). However, it is difficult to determine whether the relationship is causal or causative, as both are prevalent in areas of poor sanitation and poverty. For example, the presence of gastrointestinal parasites may lead to chronic inflammation and a reduction in the digestion and absorption of essential nutrients such as vitamin A. Conversely, a lack of vitamin A caused by malnutrition may result in reduced immune responses, and therefore a reduced ability to clear gastrointestinal (GI) parasites. Whichever of these is true, it still remains that coincidentally, people harbouring parasites are often supplemented with vitamin A, promoting a need to understand the effects of retinoic acid signalling on parasitic infections.

Some studies have been carried out to assess the effects of vitamin A on parasitic infections in humans. Indeed, re-infection of *Ascaris lumbricoides* is reduced with vitamin A supplementation of children in Mexico (6,7). This was shown to correlate with increase faecal IL-4 protein, suggesting increased Th2 responses. In general, vitamin A supplementation has also been shown to have beneficial effects on tissue inflammation in humans, demonstrated by decreased serum levels of inflammatory TNF-α and increased levels of IL-10 (8). Retinoic acid has multiple effects on innate and adaptive immunity, including promoting the differentiation of T and B cells (9,10), modulating cytokine production (11), driving Th2 responses (12,13) and inducing immune tolerance through induction of Foxp3^+^ regulatory T cells (Tregs) (14), however, the effect of vitamin A and retinoic acid signalling in specific intestinal parasitic infections is not clear. Here, we explore current knowledge surrounding the role of retinoic acid signalling in mouse models of mucosal inflammation and parasite infection. We conclude by highlighting the outstanding queries which need to be answered before we can understand fully the effects of supplementing worm-infected people with vitamin A.

## Vitamin A metabolism

**Figure 1:**
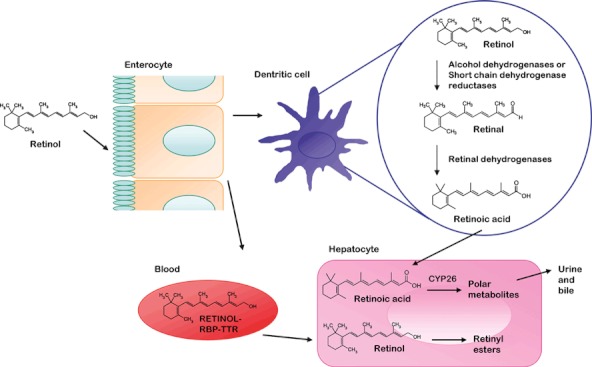
Overview of vitamin A metabolism. Vitamin A, or retinol, and precursors of retinoids in the diet (which include β-carotene and retinyl esters) are absorbed from the gut lumen. Retinol is carried around the body in the blood as a complex with transthyretin (TTR) and retinol-binding protein (RBP). Retinol is stored in stellate cells of the liver in its esterified form as a major reservoir. In some cells, including gut-associated immune cells such as dendritic cells, retinol undergoes a series of oxidation steps catalysed by alcohol dehydrogenases or short chain dehydrogenase reductases and retinal dehydrogenases, to produce retinal and the most biologically active metabolite; retinoic acid, respectively. Retinoic acid, itself, is metabolised in the liver and other tissues by the cytochrome P450 enzyme, CYP26, and its metabolites are removed from the body in the bile and urine.

Vitamin A is acquired from the diet as *all-trans*-retinol, retinyl esters or β-carotene (Figure 1). In tissues, including the gut-associated lymphoid tissue (GALT) *all-trans*-retinol is oxidised to *all-trans*-retinal by ubiquitously expressed alcohol dehydrogenases (for reviews of vitamin A metabolism see (15) and (16)). Retinal dehydrogenases (RALDHs), however, are tightly-regulated enzymes, only expressed in certain cell-types, which catalyse the irreversible oxidation of *all-trans*-retinal into the biologically active metabolite; *all-trans*-retinoic acid (ATRA), thus, making the production of retinoic acid a highly regulated process. RALDHs can be found in some gut-associated dendritic cells (DCs) and intestinal epithelial cells (IECs) (17,18), where they exist as two different isoforms; RALDH-1 mRNA is expressed in DCs from Peyer’s patches (groups of lymphoid nodules found in the small intestine) and IECs, where as RALDH-2 mRNA is expressed in DCs from mesenteric lymph nodes (MLNs), although, the physiological relevance of this expression pattern is yet to be clarified. In addition to ATRA, *9-cis*-retinoic acid, a related metabolite of *all-trans*-retinal, can be formed by either spontaneous isomerisation of ATRA, or from oxidation of *9-cis*-retinal by RALDH. Although it is controversial whether *9-cis*-retinoic acid exists *in vivo*, exogenous addition to cells shows it to have biological activity (19,20). Recently, the flow cytometric-based ALDEFLUOR assay has become a useful way to identify a cell’s potential to produce retinoic acid based on their expression of aldehyde dehydrogenases (21,22).

## Retinoic acid signalling

**Figure 2:**
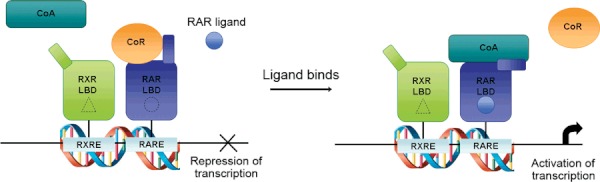
Overview of retinoic acid signalling. In the absence of an RAR ligand, the RAR-RXR heterodimer recruits co-repressors (CoR). Upon ligand-binding to the ligand-binding domain (LBD) of the receptor, a conformational change takes place that disrupts the CoR binding surface and allows co-activators (CoA) to be recruited. This, in turn, leads to the recruitment of chromatin and transcription-modifying machinery to target gene promoters and activate transcription.

Retinoic acid exerts its many effects by binding to retinoic acid receptors (RARs) and retinoid X receptors (RXRs). These receptors are intracellular nuclear hormone receptors (NHR), and each exist in three isoforms; α, β, and γ (23). RAR proteins are expressed ubiquitously and their expression is up-regulated in the presence of retinoic acid (15). Upon complexing with retinoic acid, the RARs heterodimerise with nuclear hormone receptors of the RXR family. These RAR:RXR heterodimers then go on to interact with retinoic acid response elements (RAREs) or retinoid X response elements (RXREs) within the promoters of retinoic acid-responsive genes to modify transcriptional rates (Figure 2). RXRs can form homodimers with themselves, with which *9-cis*-retinoic acid, but not ATRA, can also interact, however, *in vitro* studies have revealed that RXR has a much lower affinity for forming homodimers compared to its association with RAR to form heterodimers (24).

RXR is unique in that it is a promiscuous receptor and can also heterodimerise with many other nuclear hormone receptors. Not including RAR, the heterodimeric partners of RXR consist of the vitamin D3 receptor (VDR), thyroid hormone receptors, peroxisome proliferator-activated receptors (PPARs), liver X receptors (LXRs) and farnesoid X receptors (FXRs). These latter receptor classes are important regulators of lipid, carbohydrate and cholesterol metabolism and therefore, RXR agonists have been suggested to have potential as drugs for metabolic diseases (25). The RXR isoforms; α, β, and γ are expressed differently in mouse tissues. RXRα is abundantly expressed in the liver, kidney, spleen, epidermis and a wide range of visceral tissues, RXRβ is expressed ubiquitously and RXRγ is mostly restricted to muscles and the brain (26).

A more detailed look into the transcriptional activities of the retinoic acid receptors reveals a complexity that is not fully understood. In fact, these receptors are able to both activate and inhibit gene expression through binding of RAREs in target genes, as well as have actions of both repression and activation on other transcription factors, depending on the type and availability of retinoid ligands (27). When there are no RAR ligands readily available, un-liganded heterodimers recruit co-repressors, such as the nuclear hormone co-repressor (N-CoR) or silencing mediator for retinoid and thyroid hormone receptor (SMRT), which, in turn, recruit histone deacetylases to prevent the transcription of genes. However, binding of RAR agonists results in an active conformation of the receptor which decreases it’s affinity for co-repressors, and allows co-activators, such as CREB-binding protein (CBP) to bind and activate transcription (Figure 2). It is worth noting that RXR agonists are not able to induce the dissociation of co-repressors from the RAR-RXR heterodimer, unless RAR ligands are also present, a process named RXR ‘subordination’ (for review see (24)). This is physiologically important as it prevents confusion between several different nuclear hormone receptor signalling pathways such as retinoic acid and vitamin D3. On the other hand, some nuclear receptors, such as PPARs, are ‘permissive’ as they can become transcriptionally active in the presence of an RXR agonist alone (25).

## Laboratory models of retinoic acid signalling

Defects in retinoic acid signalling can be modelled in mice by depleting their diet of vitamin A, by genetic disruption of RAR and RXR receptors or through the use of pharmacological compounds which activate or block these nuclear hormone receptors. Vitamin A deficiency or insufficiency, achieved by feeding mice a vitamin A-deficient diet from day 14 *in utero*, as described by Smith *et al.* 1987, results in a large array of congenital defects effecting the ocular, cardiac, respiratory and urogenital systems, as well as defects of immune responses including reduced antibody responses (28,29). Furthermore, retinoic acids have been shown to rescue the effects of vitamin A deficiency in adults and during embryogenesis (30). To distinguish the individual physiological roles of RAR and RXR receptor isotypes, however, genetic mutations in mice have been created. *Rara*-, *Rarb-* and *Rarg*-null mice are all viable and only exhibit some of the characteristics of vitamin A deficiency (30). This is probably owing to functional redundancies between RARs. This theory is supported by the fact that mutants lacking two RAR isotypes, such as *Rara/b*, *Rara/g* and *Rarb/g*, die *in utero* or at birth from severe developmental defects linked to vitamin A deficiency (reviewed in (31)). Interestingly, singularly knocking out *Rxra* also results in *in utero* lethality, whilst *Rxrb*- and *Rxrg*-null mice are viable but display metabolic or behavioural defects (32). These findings are indications that RXRα is the most functionally important isotype, at least, during developmental processes (30). To overcome the limitation of *in utero* lethality, and to study the effects of these genes at later post-natal stages, spatio-temporal conditional knock-outs of RAR and RXRs have been created. The Cre-Lox system has been a valuable tool for creating conditional knock-outs of receptor isotypes in specific cell-types. For example, LysM-Cre/RXRα mice have recently been developed for the investigation of the role of RXRα signalling in cells expressing Lysosyme M, that is, macrophages and neutrophils (33,34). Pinkie mice, that have a mutation in RXRα rendering it 90% less prone to activation by ligand-binding, also exist, which allows for the study of immune responses in RXRα-deficient animals (35).

Retinoid or rexinoid are the terms applied to natural and synthetic analogues of retinoic acid that are able to mediate its biological effects through RARs or RXR, respectively. *All-trans* retinoic acid is well-established as the endogenous ligand for RAR nuclear hormone receptors, however, it remains controversial as to whether an endogenous ligand that is able to solely activate RXR *in vivo* actually exists (36). *9-cis*-retinoic acid (19), phytanic acid (37) and docosahexaenoic acid (38) have been suggested as potential natural ligands for RXR, although, these haven’t been confirmed as *bona fide* endogenous ligands to date. *9-cis*-retinoic acid is a potent activator of all RXR and RAR isotypes; so in order to further study the selective effects of these nuclear hormone receptors *in vivo*, and for potential uses in clinical applications, many synthetic retinoids and rexinoids have been developed. Some of the most widely used modulators of RAR and RXR employed in laboratory settings include Am80 (an RAR-αβ agonist) and LE540 (an RAR pan-antagonist), and HX630 (an RXR pan-agonist) and HX531 (an RXR pan-antagonist), respectively (25,39,40).

## Vitamin A in gut inflammation

Owing to the numerous effects of retinoic acid on the function of various immune cells and tissue cells previously described (16), it is perhaps not surprising that retinoic acid has also been shown to affect inflammatory processes in the gut tissue. In a spontaneous model of intestinal inflammation, vitamin A deficiency demonstrated a beneficial effect through the decreased homing of inflammatory T cells to the gut (41). Conversely, supplementation of vitamin A, as well as activation of its receptors, has also been shown to reduce intestinal inflammation. Retinoic acid can improve trinitrobenzene sulphonic acid (TNBS)-induced colitis by promoting Th2 responses (42). Studies have shown that PPAR-γ activators are able to limit the production of inflammatory mediators such as proinflammatory cytokines by macrophages and IECs through an inhibition of NFκB-driven transcription (43,44). In 2001, Desreumaux *et al.* investigated the role of the RXR-PPAR-γ heterodimer in intestinal inflammation by exploring the effects of both PPAR-γ and RXR agonists in an animal model in which colitis was induced by intra-rectal administration of TNBS. They showed that both PPAR-γ and RXR agonists were effective in reducing intestinal inflammation, demonstrated by increased survival rates, improved macroscopic and histologic scores, a decrease in TNF-α and IL-1β mRNA, and reduced NFκB binding activity in the colon; and that the agonists had synergistic effects in combination (45). In addition, genetic evidence was provided to show that PPAR-γ^+/-^ and RXRα^+/-^ mice were significantly more susceptible to inflammation than their wild-type littermates (45). Therefore, evidence exists to suggest that the activation of retinoic acid receptors could have potent anti-inflammatory effects in the gut.

Another important function of retinoic acid in immune responses is the up-regulation of gut-homing receptors. To migrate to the small intestine, lymphocytes require expression of α4β7-integrin and CCR9 (46). It has been well-established that vitamin A deficiency in rats correlates with an impaired gut-homing ability in recently-activated mesenteric lymphocytes and a decreased number of CD4^+^ T cells in the ileum (47,48). Retinoic acid alone, has been shown to be sufficient to induce the expression of α4β7-integrin and CCR9 by activated T cells, and this effect can be reversed by blocking RARs (18). The fact that of DC populations, only DCs from gut-associated lymphoid tissue express RALDH enzymes, essential for retinoic acid synthesis, further supports the essential role of retinoic acid for the imprinting of gut-homing T cells (18). However, vitamin A deficiency does not affect lymphocyte migration to the colon (48).

An important observation for infections of the gut mucosa, such as GI nematodes, is that vitamin A has been shown to contribute to barrier function of the mucosal epithelia. Studies have demonstrated that retinoic acid is able to promote crypt hyperplasia (49), and is responsible for maintaining goblet cell numbers that produce mucopolysaccharides in the small intestine (50). Furthermore, vitamin A deficiency results in a lengthening of the duration of the cell cycle in jejunal crypt cells, thus slowing down their possible differentiation into enterocytes or goblet cells (51,52). Since vitamin A is also absorbed through the gut mucosa, these effects may be important in promoting its own uptake into the body. The role of retinoic acid signalling in maintaining the mucosal epithelium in the large intestine remains largely unexplored, however, more recently, retinoic acid has been shown to increase the expression of genes involved in intracellular tight junction formation *in vitro* (53), which also may promote epithelial barrier function. These investigators also showed that depletion of retinoic acid in the mucosal epithelium was proportional to a decrease in barrier function *in vivo*, suggesting that the availability of retinoic acid in the mucosal epithelium is important for determining barrier integrity (53).

## Retinoic acid signalling in mouse models of intestinal parasitic infection

The four major mouse models of gastrointestinal nematode infection are: *Trichinella spiralis*, *Trichuris muris* and the hookworms *Heligmosomoides polygyrus (bakeri)* and *Nippostrongylus brasiliensis*. Other parasitic infections with lifecycle stages that occur in the gastrointestinal tract include the protozoan parasite *Toxoplasma gondii* and blood fluke *Schistosoma mansoni*. Very few studies have investigated the effects of vitamin A on host resistance to GI nematode infections in animal models, despite delivery of vitamin A to humans infected with intestinal parasites. The lack of investigations is also surprising given the importance of dietary absorption of vitamin A through the niche of these parasites; the GI tract, and also since vitamin A has so many well-established effects on the immune responses (for review see (16)). Here, we review the little work that has been carried out and discuss how the known effects of vitamin A signalling could impact parasitic infections.

### Retinoic acid and T Helper cell responses

In general, GI nematodes induce protective Th2 responses in the host, which are characterised by the production of the cytokines IL-4, IL-5, IL-9 and IL-13, the antibodies IgG1 and IgE, and accompanied by eosinophilia, basophilia, mastocytosis, and the expansion of alternatively-activated macrophages (for review see (54)). The development of these responses allows for expulsion of the parasite. *Trichinella spiralis* infection is an acute infection of mice with a life cycle stage in the small intestine. Expulsion of this parasite is dependent upon the generation of a Th2 response, in particular, the presence of mast cells (55,56). Investigators have shown that infecting vitamin A-deficient mice with *T. spiralis* has no effect on the number of larvae produced in the small intestine or the muscle larvae burden at 5 weeks post-infection, although, at day 15 post-infection only vitamin A-deficient mice still harboured enteric parasites, while vitamin A-sufficient animals had cleared the infection in the intestine (57). These investigators also showed that vitamin A-deficient mice had reduced numbers of B cells secreting parasite-specific IgG1 antibodies, reduced eosinophilia in the bone marrow, as well as more mesenteric lymph node cells secreting IFN-γ and significantly less secreting IL-4 and IL-5, consistent with a delay in expulsion of the enteric stage of the parasite (57). Further studies have shown that the shift from Th2 to Th1 responses during *T. spiralis* infection of vitamin A-deficient mice, is due to faster production of the Th1 cytokine IFN-γ by cells, antigen presenting cells (APCs) inducing more IFN-γ release and fewer cells producing the Th2 cytokine IL-5 (58).

The observations of increased Th1 responses and decreased Th2 responses during *T. spiralis* infection in the absence of vitamin A are in agreement with previous studies investigating the effects of retinoic acid. Indeed, vitamin A deficiency in rats correlates with decreased Th2 responses, indicated by decreased IgA, IgE and IL-6 levels (59). Further research has shown that retinoic acid induces Th2-cell promoting transcription factors, such as GATA-binding protein 3, macrophage-activating factor and STAT6 (12). RXRα defective pinkie mice have an exaggeration of Th1 but loss of Th2 responses (35). Taken together, this evidence suggests that retinoids have an important role in Th2-mediated immune responses, which may have important implications in the inflammatory response to parasite infections such as *T. spiralis*. In contrast, however, recent experiments using the Th1 driving-intracellular parasite *T. gondii* have shown that retinoic acid is required for the generation of Th1 and Th17 responses in the gut, post-oral infection with this parasite (60). These investigators showed that vitamin A insufficiency caused decreased IFN-γ production by MLN-derived CD4^+^ T cells, as well as decreased expression of α4β7 integrin, that is, gut homing molecule expression, on these cells in response to *T. gondii* infection (60). It was also observed that Th17 cells from vitamin A insufficient *T. gondii*-infected mice had a reduced expression of Ki67 and were therefore less proliferative compared to vitamin A sufficient controls. Furthermore, all these effects were restored by addition of retinoic acid (60). These findings were surprising given that previous studies have shown that vitamin A supplementation has the opposite effect, blocking the production of Th1 cytokines *in vitro* and *in vivo* (11,61). Moreover, retinoic acid can dampen the expression of the Th1-cell associated gene, T-bet (12). Hall *et al.* (60) suggest that their data may conflict with *in vitro* work previously carried out due to the addition of retinoic acid to already established effector/memory responses in *in vitro* cultures, whereas, their model of vitamin A insufficiency from birth demonstrates the effect of retinoic acid on the development of immune responses to the parasite. Therefore, retinoic acid may act to aid T cell activation during the early stages of immune responses, but regulate effector responses at later stages. Hall *et al.* (60) propose that this could be a strategy to produce effective initiation of immune responses but prevent excess damage to the host during chronic infections. A mechanism for this process has not yet been elucidated, although, it could be owing to changes in expression of RALDHs during infection/inflammatory responses, or changes in the expression of retinoic acid-responsive nuclear hormone receptors, which could lead to the activation of differential signalling pathways inside T cells, for example.

Hall *et al.* (60) demonstrate that the lack of Th1 responses to *T. gondii* infection in vitamin A insufficient animals leads to increased parasite burdens in these mice. This is true of both lamina propria and systemic burdens following oral infection, as well as systemic and peritoneal parasite burdens following intra-peritoneal infection (60). These findings show not only the beneficial effect of retinoic acid signalling during *T. gondii* infection, but also that vitamin A metabolites are important systemically and not just in immune responses of the gut-associated lymphoid tissue. Other experiments investigating the effect of retinoic acid on a kidney fibroblast cell-line infected *in vitro* with *T. gondii* tachyzoites also suggested that retinoic acid had a protective effect in this infection, as there was a reduction in the number of infected cells and the number of larvae in the cells cultured in the presence of retinoic acid compared to control cultures (62). More research is needed, however, to establish whether administration of retinoic acid *in vivo* is sufficient to reduce/protect against *T. gondii* infection in mice and humans.

### Retinoic acid and regulatory T cells

It is now well-established that retinoic acid is important in driving TGF-β dependent differentiation of FoxP3^+^ regulatory T cells (17,63,64). Moreover, MLN-DCs from vitamin A-deficient mice have a reduced capacity to induce FoxP3^+^ cells (65). *H. polygyrus (bakeri)* is a natural trichostrongylid nematode infection of the mouse, whose lifecycle takes place entirely in the GI tract (66). Primary infections with *H. polygyrus (bakeri)* result in chronic infection that is modulated by Th2 responses and characterised by increased IgE, IgG1 and eosinophilia, however, in this infection, regulatory T cells also play an important role. Indeed, *H. polygyrus (bakeri)* produces its own TGF-β mimic, which induces TGF-β-producing CD4^+^ T cells, and therefore also Tregs (67). Other helminths are also known to induce Treg differentiation. Work using *S. mansoni* soluble antigen preparations has shown that one of these antigen proteins is able to drive the development of Foxp3-expressing T cells in a retinoic acid-dependent manner (68). This retinoic acid-driven mechanism may be advantageous for parasites to suppress immune responses, and promote their own survival. Therefore, retinoic acid signalling may be a potential target for the treatment of parasitic infections, especially those dependent upon regulatory responses in the host.

Disappointingly, little work has been carried out investigating the effect of vitamin A signalling on *H. polgyrus (bakeri)* infection, however, recently, a new population of CD11c(lo) nonplasmacytoid dendritic cells (DCs) that expand during this parasitic infection have been identified. These DCs are highly tolerogenic and are important in inducing regulatory (FoxP3^+^) T cells, as depletion of these cells results in decreased numbers of Tregs. Furthermore and importantly, the generation of Tregs by CD11c(lo) cells was retinoic acid-dependent, as induction of CD4^+^Foxp3^+^ cells was reduced significantly by addition of the RAR antagonist LE540 to *in vitro* cultures (69). The potential of gut DCs to produce retinoic acid has recently been shown to lie within the CD103^+^ population (70). An investigation into the role of CD103 in the immune responses to the caecal-dwelling *T. muris* parasite has shown accentuated T cell responses post-infection in CD103^-/-^ mice (71). Given that tolerogenic CD103^+^ DCs have been found to be important retinoic acid producing cells in the gut mucosa (21), it was suggested that the increased T cell responses could be due to a lack of retinoic acid signalling and therefore a reduced induction of Tregs, although, no change in Treg populations were observed in the gut or MLNs of CD103^-/-^ mice in this study (71). Therefore, retinoic acid signalling may have a role in directing the regulatory and suppressive properties of the immune response to *H. polygyrus (bakeri)* and *T. muris* infection through actions on DCs.

Interestingly, Hall *et al.* show that vitamin A insufficient mice infected with *T. gondii* actually have increased numbers of Foxp3^+^ Tregs as opposed to decreased numbers as wisdom would dictate (60). This may be a consequence of decreased Th1 and Th17 responses observed in this investigation (60), as discussed earlier, although this is not clear. The complex and confusing nature of retinoic acid signalling as demonstrated here, reveals the need for further research in this area.

## Effects of retinoic acid on parasite vitality

Any discussion concerning the supplementation of the diet of animal models of infection with vitamin A ligands should also include effects on the parasite, rather than just on host immunity. Some intestinal parasites are able to secrete retinol-binding proteins that can sequester retinoic acid close to the parasite’s niche. Studies have shown that the filarial parasite *Onchocerca volvulus* secretes a protein, named Ov-FAR-1, into the tissue surrounding it, which has high-affinity binding sites for retinol and fatty acids and therefore may contribute to the ‘river blindness’ caused by this parasite, through a reduced availability of retinol in the eye (72). Other investigations have revealed that *Brugia malayi* is able to take up radio-labelled retinoic acid. Further, the retinoic acid was localised in high concentrations at early and late embryonic stages, suggesting a role for retinoic acid in its growth and development (73). Detailed analysis of the ABA-1 allergen of the pig nematode *Ascaris suum* has also demonstrated retinol and retinoic acid binding capabilities (74). We can speculate that this sequestration of retinoic acid may be an immunomodulatory mechanism used by the parasite in order to locally deplete retinoic acid from the host and therefore alter the local immune response, possibly by decreasing Th2 responses, and thus favouring its own survival, although this is yet to be confirmed. Interestingly, some parasites, such as *S. mansoni* have been shown to have RXR-like receptors which seem to be important for female gene expression (75). Therefore, it would be interesting to assess whether retinoic acid can affect worm viability or fecundity (especially given the link to female gene expression). Currently, it is unknown whether gastrointestinal nematodes produce retinol-binding proteins or are able to respond to retinoic acid, although, genomic sequencing of these parasites will hopefully help to answer these questions.

## Conclusion

Gastrointestinal parasites have an astonishing prevalence across the globe and are responsible for significant morbidity through the development of intestinal inflammation. Immune responses can govern susceptibility to intestinal parasites and have been extensively studied in the mouse models of these infections, although these immune responses are still not fully understood. Vitamin A metabolites, such as retinoic acid, have been shown to modulate the Th1/2 balance in favour of the parasite-protective Th2 immune response, as well as increase the generation of gut-homing Tregs important for dampening inflammation. Therefore, it is perhaps surprising that so little work has been carried out in this field, especially given the link between the prevalence of vitamin A insufficiency and parasitic infections in humans.

The literature surrounding retinoic acid signalling during gut inflammation and infection is confusing and inconsistent, and many questions still remained unanswered. For example: Is vitamin A pro- or anti-inflammatory? Does it behave differently in the context of qualitatively different (Th1 or Th2 biased) inflammation? Is it unbiased in its control of IBD? Is the sequestration of retinoic acid by the parasite an evasion mechanism? Indeed, vitamin A deficiency has been shown to have a beneficial effect in a spontaneous model of intestinal inflammation by decreasing the homing of CD4^+^ T cells to the gut (41). In opposition to this, dietary supplementation with retinoic acid can also reduce intestinal inflammation in a model of TNBS-induced colitis by enhancing Th2 responses (42). Vitamin A-deficient mice infected with the Th2-inducing GI nematode *T. spiralis* display increased Th1 responses and diminished Th2 responses compared to vitamin A sufficient controls (58). Conversely, vitamin A-deficient mice have impaired Th1 and Th17 responses to the Th1-inducing parasite *T. gondii*, and this can be rescued by the administration of retinoic acid (60). One possibility regarding these inconsistencies is that retinoic acid is unbiased in its control of immune responses and serves to regulate on-going immune responses, whatever they may be, although the mechanism of this is not yet clear. In accordance with this, Dawson *et al.* show that the administration of physiological doses of retinoic acid to pigs with *Ascaris suum* infection results in the diverse augmentation of Th1, Th2, Treg and inflammatory responses, with seemingly no bias towards either ‘arm’ of the immune response (76). The concentration of retinoic acid may be a factor in determining the outcome of retinoic acid signalling. For example, reports have shown that high concentrations of retinoic acid can block the differentiation of Th17 cells (63), however, low concentrations of retinoic acid seem to be essential for Th17 cell differentiation (77). Furthermore, in the *Ascaris suum* pig model of helminth infection, a dose-dependent activity of retinoic acid was reported, with the most active doses being surprisingly lower than those generally used in rodent experiments (76). Studies have shown that vitamin A absorption is decreased in children with intestinal parasite infections (78). Therefore, perhaps, the presence of a higher burden of parasites in the gut results in more disruption of the intestinal epithelium and a decrease in the absorption of vitamin A that can be converted to retinoic acid. Thus, differential endogenous concentrations of retinoic acid may be available in low and high-burden models of GI parasite infection. The variation of immune responses that occur to different intestinal parasites also adds a lack of clarity to the role of retinoic acid signalling during these infections. Further research in this field is important to establish the effects of administering vitamin A to humans infected with GI parasites that is currently occurring.

## Abbreviations

ADH: Alcohol dehydrogenases
APC: Antigen presenting cell
ATRA: All-trans-retinoic acid
CBP: CREB-binding protein
DC: Dendritic cell
FXR: Farnesoid X receptor
GALT: Gut-associated lymphoid tissue
GI: Gastrointestinal
IEC: Intestinal epithelial cells
LXR: Liver X receptor
MLN: Mesenteric lymph node
N-CoR: Nuclear co-repressor
NHR: Nuclear hormone receptor
PPAR: Peroxisome proliferator-activated receptor
RALDH: Retinal dehydrogenase
RAR: Retinoic acid receptor
RARE: Retinoic acid response elements
RBP: Retinol-binding protein
RXR: Retinoid X receptor
RXRE: Retinoid X response element
SMRT: Silencing mediator of retinoid and thyroid hormone receptor
TNBS: Trinitrobenzene sulphonic acid
Treg: Regulatory T cell
TTR: Transthyretin
VDR: Vitamin D3 receptor
